# Supplementary material for: Global and gene-specific translational regulation in Escherichia coli across different conditions
Source: PLoS Comput Biol. 2022 Oct 20;18(10):e1010641. doi: 10.1371/journal.pcbi.1010641 (PMC9624429; doi:10.1371/journal.pcbi.1010641)
Supplement: Attachment — Submitted filename: Response_to_Review_20220812.docx [file pcbi.1010641.s004.docx]

**Response to reviewers**

**Reviewer #1**:

The correlation between transcription output and translation output remains controversial but is of great importance and broad impact in molecular biology and systems biology (including the application of synthetic biology). Combing RNA-seq and Ribo-seq, Zhang et al use E. coli as a model system and quantify the mRNA level and translation efficiency (TE) for individual genes in various poor nutrients.

As pointed out by Gene Wei Li (Curr Opin in Microbiol, 2015, 23:66-71) (the author could cite this paper), the major determinant of TE is still largely unknown, e.g., RBS (SD sequence) has been found to be not quite related to TE. Therefore, the issue of TE regulation is a very important open direction to investigate. This paper provides very useful large-scale TE information of bacteria to guide future theoretical quantitative studies and synthetic biology applications.

The paper is generally well written and is of broad interest for the community of Plos Comput Biol. I have just a few minor comments for the authors, mostly for clarification.

We thank the reviewer for all the thoughtful comments. An appropriate citation to the paper of Gene Wei Li et al. 2015 has been added.

1. Line 169: The definition of RTE: the ratio of the transcript abundance of the ribosome profiling to relative mRNA level. I suggest making it more clear here: first introduce the definition of TE: the rate of protein production per mRNA (the translating ribosome number per mRNA). Then claim that here TE is represented by dividing rate of synthesis of each protein (ribosome density by ribosome profiling) by the corresponding mRNA levels as measured by RNA-seq, namely RTE.

We thank the reviewer for this suggestion. We agree that the definition of RTE is essential to this work and deserves clarification. We have added the common definition of translation efficiency (TE) in line 179 (with reference to Gene-Wei Li, PMID: 25636133), as “Translation efficiency (TE) has conventionally been quantified as the rate of protein production per mRNA (the translating ribosome number per unit length of a mRNA). Here, a version of TE is obtained by dividing the rate of synthesis of each protein (ribosome density from ribosome profiling) by the mRNA levels from RNA-Seq under the corresponding condition; we call this quantity the “relative translation efficiency” (RTE, see Methods for details).” We believe this makes our definition of relative translation efficiency (RTE) clearer.

2. Figure 2C: please provide the definition of actual RTE and random RTE. Not quite clear here.

We thank the reviewer for this suggestion. We have added a more transparent definition of actual RTE and random RTE in the legend of Figure 2C, as “(blue bars – Actual RTE: original RTE from RNA-Seq and Ribosome profiling; gray bars – Random RTE: the RTE values for each gene were randomly scrambled among the 12 conditions to obtain the randomly ordered RTEs, which was considered as the null distribution, see methods and S1 Text for details)”. Also, more detailed descriptions are in Methods and S1 Text, including line 725-730 in the main text and item 1 (Theoretical analysis for the distribution of correlation coefficients between mRNA level and randomly scrambled RTE) in S1 Text.

3. In Figure 2D and 2E (also such as Figure 3C, 3D and related places), I suggest also showing the data of ribosome density (from ribosome profiling) at the middle between mRNA and RTE. The ribosome density is the total output of protein production rate of a specific gene. You have RTE=ribosome density/mRNA, correct?

This is an excellent suggestion. Figure 2D-E and Figure 3C-D have been revised to include the ribosome footprints in the middle panel. As to the question about RTE definition, yes, we have RTE=ribosome density/mRNA. In a more detailed definition, ribosome density is obtained from the footprinting counts on a particular gene normalized by the length of the gene and by the total footprinting, while mRNA level is quantified by normalized counts from RNA-Seq.

4. About the point of rare codon issue, according to Li et al 2012 Nature, codons usage seems not impact in vivo translation rates (in rich medium). Does the author note sth different in poor nutrient conditions? If so, this could be another useful information to show and make some useful discussions.

We thank the reviewer for this question. The connection between codon usage and translation rate is one of the key questions in this field. According to Li et al 2012 Nature, rare codons do not affect translation efficiency in nutrient-rich media. However, bacteria in nature must adapt to changing environments, and rapid exponential growth, such as in lab conditions, is uncommon. Studies have reported that under stresses such as nutrient limitations and oxidative stress, translation efficiency is sensitive to codon-usage-mediated tRNA dynamics (O'Shea et al. Cell 2014, PMID: 25416955, Mata et al. NAR 2021, PMID: 33313903). However, the details of how translation efficiency changes under stress remain unclear (Dai et al. Cell Mol Life Sci 2020, PMID: 31552449). It has been proposed that stress-response genes are more prone to use rare codons, and the change of the corresponding tRNA content for rare codons under stress conditions causes the up-regulation of the translation of these stress-response genes (Babu et al Sci Signal 2018, PMID: 30181241).

In our study, in order to further quantify the influence of codon frequency on the gene expression patterns, we added a comparison between the top 200 and bottom 200 genes in their mRNA levels (Fig J in S1 Text). The codon frequency of the two clusters of genes was negatively correlated, such that genes with high mRNA levels tend to contain non-rare codons, while genes with low mRNA levels tend to contain rare codon. Furthermore, the relative translation efficiency of the two types of genes differs in sensitivity to nutrient limitations (Additional Fig. 1, shown below). We noticed that the bottom 200 genes in mRNA levels, i.e. the genes tending to containing rare codons, are more sensitive to nutrient limitations (Additional Fig. 1A). These results support the idea that codon usage is involved in translational regulation, especially under stress.

We have added a discussion of this point in the manuscript (line 559-562).

Additional Figure 1. (A) The RTE distribution of 200 genes with the lowest mRNA levels, which tend to contain rare codons (Fig J in S1 Text). Different colors represent different nutrient conditions and growth rates. (B) The RTE distribution of 200 genes with the highest mRNA levels, which tend to contain non-rare codons. Different colors represent different nutrient conditions and growth rates.

////////////////////////////////////////////////

**Reviewer #2:**

Zhang et al attempts to uncover the gene-specific regulation at the level of transcription and translation in response to carbon, nitrogen and phosphate limitations. It is a timely question, the authors try to tackle different points and probably it is not clear (at least to me) what the main message of this work should be. The main result that has been highlighted is that “random forest model suggests that codon usage partially predicts a gene’s cross-condition variability in translation efficiency”. However, this is just a small part of the manuscript, which arrives at the end and that could be better discussed/integrated.

We appreciate the reviewer's insightful comments. We have reorganized the text and figures to better convey this work's main message.

1. The introduction is rather well written, however I believe that there is some important literature missing. For instance, when the authors discuss how “E. coli cells are known to cope with different nutrient limitations by changing ribosomal synthesis and usage strategies” (line 78), they should probably cite the pioneering works by Schaechter or Bremer&Dennis, or more recent publications (e.g. PMID: 21097934); there are several “theoretical models … developed to provide an integrative picture of translational regulation”, other than the cited ones (e.g. PMID: 24688632, 29694095, 27591251, 29448386). The literature about the role of secondary structures on the efficiency of translation is vaste (e.g. 31760691), the authors could probably explore this field a bit more in depth.

We thank the review for this suggestion. We have added several citations relevant to “*E. coli* cells cope with nutrient limitations by changing ribosomal synthesis and resource allocation strategies” (PMID: 21097934), and theoretical models about translation processes (PMID: 24688632, 29694095, 27591251, 29448386) in lines 78 and 88. The effect of mRNA structure on translation efficiency remains a contentious area of active research. Largely unknown are the structural elements that influence translation efficiency, especially in response to stress. We have accordingly modified this section in the Introduction and cite the studies on mRNA structure in translational regulation (PMID: 31760691, 28918899) to reference this field in more depth in lines 93-96, as “However, others suggested that mRNA structure itself is a key factor in translational regulation, which contributes to the rapid adaptation of changing environments by dynamically folding in response to several signals, such as temperature changes, ligands, regulatory proteins, and small RNA.”.

2. One of the main questions is the relation between this manuscript and other recent works from other groups (for instance Mori et al. PMID: 34032011), where data is available also for a broader range of growth. Can the authors clarify the novelty of their work?

We thank the reviewer for this question, which has spurred us to clarify the novelty of our work. In the paper from Mori et al., the proteome of *E. coli* under multiple growth conditions was quantified at an absolute level using mass spectrometry, providing valuable resources for multi-omics studies of gene regulation and metabolic control in *E.* coli. However, in this paper from Mori et al., the ribosome profiling technique was used only as a calibration of the absolute protein abundance, and due to the cost of the technique, the authors did not perform ribosome profiling experiments under multiple conditions.

Ribosome profiling can help to quantify translation efficiency and elucidate translational regulation processes and translational events at the genome-wide level, while the protein abundance obtained by mass spectrometry reflects the result of all levels of regulation, including the translational level. Therefore, our ribosome profiling data under multiple nutrient limitations are complementary, not redundant, to the absolute quantitative proteomics from Mori et al. In the future, multi-omics studies will benefit from a fusion of transcriptomics, translatomics, proteomics, and metabolomics data. For now, our study has provided the most complete study to date of Ribo-seq combined with RNA-seq data in *E. coli* regarding nutrient limitations. As such, we believe that it will also provide a valuable resource for future studies.

In addition, compared to the batch culture in other studies (for instance Mori et al. PMID: 34032011), we used chemostats to precisely control the growth rate of bacteria while applying nutrient limitations. This allowed us to analyze the changes in translation efficiency in the dimensions of different growth rates or nutrient limitations separately, making our data unique. In conclusion, we systematically elucidate the bacteria translational regulation in response to nutrient limitations both at the global level of ribosome usage and at the genomic level of translation efficiency, which provides novel insight into translational regulation in response to stresses.

We again thank the reviewer for this question and we have now more clearly emphasized the importance of our data as a resource in the Abstract of this paper, in line 41. Moreover, we have cited the paper from Mori et al. in the introduction and acknowledged its value in line 82. We also pointed that “clarifying the detailed process and mechanisms of translation regulation requires the integrated analysis of multiple omics, including transcriptomics, translatomics, and proteomics” in line 83.

3. Methods are often taken from a previous publication of the group (ref 18). However, it is useful to explain how the measurements are done and when the values are not directly experimentally measured and when are inferred by fitting the model (e.g. elongation rates).

We appreciate this suggestion from the reviewer. We have accordingly modified the Methods and elaborated in greater detail on the experimental measurements and parameters inferred from the model. In lines 603, 613, and 656 of the Methods, currently it states “Experimental measurement of total RNA”, “Experimental measurement of total protein”, and “The total number of ribosomes was calculated based on the experimentally measured parameters”

In a brief summary, the growth rate, total RNA, total protein, and elongation rate were all experimentally measured. The fraction of ribosomes was obtained from polysome profiling. The total number of ribosomes was inferred from

$$R_{t}= V_{c}\cdot C_{p}\cdot RPR\cdot\frac{f_{\text{r}}}{m_{r}}$$

where the $V_{c}$ is cell volume (m^3^) (PMID: 26443740), $C_{p}$ is concentration of proteins (g/m^3^) (PMID: 2200929), $RPR$ represents RNA-to-protein ratio, $m_{r}$ is the mass of the rRNA component of a ribosome (g) (PMID: 14681416), and $f_{\text{r}}$ is the fractional mass of rRNA among total RNA.

4.Line 132: The free ribosome pools decreased as growth rate increased… how is that measured? To my understanding there is no experimental way to determine the amount of free (or bound) ribosomes. It is possible to use polysome profiles (as more or less said in the caption of Figure 1) but the way the profiles can be analysed remains arbitrary (70S are considered free or bound? What about 30S and 50S?

We thank the reviewer for raising this point. Indeed, previously it was difficult to directly distinguish the free 70S peak and bound 70S peak via normal polysome profiling technique, because of the relatively small mass of mRNA compared to ribosomes. In one of our previous works (Li et al. Nature Microbe 2018, PMID: 30038306), a novel method was introduced to distinguish free and mRNA-bound 70S monosomes. In this method, the differential sensitivities of free and mRNA-bound 70S monosomes to high potassium levels (170 mM) were used (PMID: 4558708). High potassium causes free ribosomes to shift to a lower density but does not shift the density of mRNA-bound monosomes. Therefore, in the process of polysome profiling, 100 mM NH_4_Cl was replaced with 170 mM KCl. Then the partially overlapped peaks (free 70S, and bound 70S) were fitted by two Gaussian distributions to quantify their abundances. In the previous study (PMID: 30038306), this method effectively extracted the fraction of free 70S and bound 70S according to control experiments. We adapted the method in this study. More detailed experimental methods are given in line 625-655.

5. Panels C-F of figure 1 are difficult to read. At least the authors should give the range and draw a few axis ticks. Labels in panel A can be improved…

We thank the reviewer for this suggestion. We have marked the axis ranges of all the radar plot in Figure 1C-F and modified the labels in Figure 1A.

6. Why do the authors not investigate TE and the fraction of transcript of the gene of interest with respect to the total amount of mRNA? I imagine that this would be more meaningful, and probably one can avoid possible normalisation problems (PMID: 32284352). I may be missing something, but it seems to me a more robust analysis to understand the tradeoff of transcription (mRNA) and translation (RTE).

We thank the reviewer for this suggestion. According to the definition in our study, RTE was calculated as

$$[RTE]=\frac{[footprinting counts]/[gene length]}{[relative mRNA level]} ,$$

where the footprinting counts were normalized by the total footprinting counts in that experiment. The ratio of footprinting counts to gene length reflects the relative ribosome density. The relative mRNA levels were also normalized by the total mRNA counts and by gene length as reads per kilobase million (RPKM) (lines 714-721). Therefore, both the footprints and mRNA levels were normalized before being used to calculate RTE. The RTE represents the relative occupancy of ribosomal resources devoted to translation, rather than the absolute protein production rate per mRNA molecule.

For the normalization problems mentioned in the paper (PMID: 32284352), we have checked our experimental processes and data quality. According to the paper, errors may be introduced when comparing samples with different preparation protocols, tissues with different mRNA distribution, and samples from different RNA compartments. In our study, we minimized these problems by performing the same preparation and sequencing protocol on 36 samples. In addition, the data quality in our study has been illustrated in the Fig A-B in S1 Text and described in the results. There were very high correlations of mRNA expression levels between the biological replicates (Fig A in S1 Text). And the mRNA levels of all samples followed similar distribution after normalization, indicating that the samples could be compared in our study. Therefore, we believe that our current normalization methods avoid most of the artifacts, and accurately depict the patterns of gene expression in response to environmental conditions.

7. Figure 2: “Mean levels were taken as the average of all 12 conditions (Table 1)”. Why? Would not be more useful to do the same for each condition (or for at least each growth rate).

We thank the reviewer for this question. Figure 2B was used to exhibit the correlation between mRNA level and RTE across different genes. So, the average expression level under all 12 conditions for each gene is most suitable to illustrate differences between genes, independent of specific conditions. However, we agree that it is also informative to show the correlation between mRNA level and RTE for each condition, and we have performed these analyses (Fig N in S1 Text). The results show that the overall positive correlation between mRNA level and RTE across different genes remains consistent across all 12 conditions.

8. Figure 2D-E: Scatter plots would be more useful (and one can color the points with the growth rate and conditions with different symbols).

We thank the reviewer for this suggestion. We have added the scatter plots of the top 5 genes with negative or positive correlations between mRNA level and RTE in the S1 Text (Fig E in S1 Text). In the main text, we now show the ribosome footprints as the middle panel in Figure 2D-E and Figure 3C-D.

9. Line 278: “almost all of their RTE…” Can the authors provide a percentage?

We thank the reviewer for this suggestion. We have modified the main text: “83.3% (40/48) of their RTE fold changes were also greater than 1” at line 297.

10. About the upregulation of RTE and mRNA, I am slightly worried that this results could be an artifact of normalisations of the data (the more mRNAs one has, more ribosome footprints etc etc). Can the authors comment on that and check that this is not the case?

We thank the reviewer for this question, to which we also devoted great attention during our analysis. Indeed, it is absolutely correct that for the sequencing technique used, the more mRNA there is of one gene, the more ribosome footprints that gene will have. Consequently, the data from ribosome profiling alone cannot fully reflect the changes in translation efficiency. That's why we calculated relative translation efficiency (RTE). RTE, defined as the ratio of the transcript abundance obtained via ribosome profiling to relative mRNA level, represents the ribosomal density on a single mRNA of each gene. Mathematically, the ribosome footprints were normalized by by gene length and by the total footprinting counts. The mRNA counts were correspondingly also normalized by gene length and by the total mRNA counts, as reads per kilobase million (RPKM). Then the ratio of normalized footprints to normalized mRNA level was used to quantify RTE.

Practically, polycistrons provide good test cases to confirm that the changes of RTE are not artifactually dependent on mRNA level, gene length, etc. To test whether RTE truly reflected differences in translation between genes, we analyzed the expression pattern of genes from the *dusB-fis* operon and the F0F1 ATP complex, which are two typical cases of genes controlled at the translation level with similar mRNA abundances (lines 190-200). The RTE in our study accurately reflected the changes of translation efficiency (Fig C in S1 Text), consistent with previous studies by Li et al (PMID: 24766808, 28139975) that quantified absolute translation efficiency. These results confirm the reliability of our definition of RTE in quantifying translational differences among genes.

11. It is somehow difficult to understand the main message of Figure 5. The authors should try to clarify it in the main text (and maybe chose a different way to show the data?). The same it is also true for panels G and H of the same figure: it is not clear why the authors decide to talk about the codon usage at this point (and not wait till the following section).

We thank the reviewer for this suggestion, and we have rewritten the main text in this section and rearranged the figures to make the logical flow more clear: Before Figure 5, the results we present reveal a diverse range of translational regulations of genes in response to different nutrient limitations. Therefore, we wanted to explore whether translation regulation was associated with some general factors. As genes involved in the uptake of C, N, and P all exhibited specific upregulation in RTE under corresponding nutrient limitations, we suspected the existence of one class of “highly responsive in RTE” genes: those that changed RTEs across conditions, thus exhibiting large RTE variance. Therefore, we turned our attention to the RTE pattern of genes, and analyzed genes with different RTE variances. These results, which are shown in Figure 5D-E, suggest the RTE patterns are associated with gene function. According to the reviewer's helpful suggestion, we moved our analysis of codon usage to the next section (Figure 6-7) to make the overall logic clearer.

12. Figure 6A. Other than the random forest, the authors could randomly choose 200 lowly expressed genes and 200 highly expressed genes (without looking at RTE) and try to do the same plot.

We thank the reviewer for this suggestion. We have added Fig J in S1 Text to show the mean codon frequencies for the top 200 genes with the highest mRNA levels and those for the bottom 200 genes with the lowest mRNA levels (Fig J in S1 Text, Spearman’s rank correlation coefficient -0.30). The nine rarest codons in *E. coli* (AGA, AGG, ATA, CCC, CGA, CGG, CTA, GGA, and TTA, according to PMID: 16703415) were enriched in genes with low mRNA levels (Fig J in S1 Text). However, the nine rarest codons showed no obvious bias in the two sets of genes with largest and smallest RTE variance, and none of the typical four genes in Figure 6B is rare codon. This suggests that the RTE changes mediated by codon usage in our study are distinct from the low translation efficiency mediated by rare codons.

/////////////////////////////////////////////

**Reviewer #3:**

In this work, the authors analyzed the translational regulation which might be a major cause that led to the well-known discrepancy between mRNA and protein levels. They first showed that E.coli cells achieved the same growth rate at different nutrient conditions by differentially regulate three ribosome features. The authors then combined ribosome profiling and RNA-seq to quantify the relative translation efficiency (RTE). By analyzing the pattern and correlation of mRNA and RTE levels, the author assessed the gene-specific and condition-specific translational regulation. They further demonstrated the relationship between codon frequencies and translational regulation patterns. These results would be important to help people understand the translational regulation of genes and help bridge the gap between mRNA and protein. Here are some comments and revisions that might need to be made:

We thank the reviewer for all the thoughtful comments.

1. In figure 1A, data points from the same condition but at different growth rate are connected by straight lines, which seem to suggest linear relationship between the mRNA-protein ratio and the growth rate. However, with only two different growth rate levels, there are not enough data points to make assumptions on the linearity.

We thank the reviewer for this comment. The existence of an empirical linearity between mRNA-protein ratio and growth rate was established by a significant phenomenological study from Terence Hwa’s group, Scott et al. (PMID: 21097934). The robust empirical correlation of the RNA/protein ratio with the growth rate from Scott et al. suggested underlying constraints of cellular resource allocation, and has impacted a large number of following works in *E. coli* metabolism (e.g., PMID: 24084808, 25695966, 30038306). In our previous study, also using a chemostat setting as in this work (PMID: 30038306), and under the same type of nutrient limitation, there was a linear correlation between the growth rate and RNA-to-protein ratio according to four different growth rates. To highlight this now well-established relation, we have added text with appropriate citations in line 141: “All other conditions are located on a single line of R/P ratio versus growth rate (Fig. 1A), consistent with the well-established linear relationship between growth rate and RNA-to-protein ratio in previous studies[18, 39, 40].”

2. The colors representing free 70S monosomes, mRNA-bound 70S monosomes and mRNA-bound polysomes are not correctly noted in the figure legends of figure 2B. For example, free 70S is represented in dark color in figure 2B. But the figure legends says that it is represented in white.

We thank the reviewer for the careful reading. We have corrected this error in the legend of Figure 1B.

3. A positive correlation of coefficient of determination of 0.3 between mean mRNA and RTE was observed when cross-gene relationship was analyzed. However, more negatively correlated mRNA and RTE pairs were identified when the cross-condition correlation analysis was performed. Is there any explanation for the conflicted observations in the cross-gene and cross-condition correlation analysis?

Additional Figure 2. The illustration of cross-gene and cross-condition correlation analysis. Each point represents a gene.

We thank the reviewer for this comment. In fact, in the cross-gene and cross-condition correlation analysis this apparent conflict is actually not inconsistent. The mRNA-RTE correlation of single genes across conditions can vary a lot. But, when one puts all the genes together and calculates the mRNA-RTE correlation across different genes, there is still an overall positive correlation (Additional Figure 2). In extreme cases, the combination of multiple negatively correlated data sets may show an overall positive correlation. (This phenomenon in statistics is “Simpson's Paradox”.)

In fact, the cross-gene correlation and the cross-condition correlations have long been the major focus in this field. In our study, under each condition, there was a positive correlation between mRNA level and RTE across different genes, which reflects inherent translational differences between genes that synergize with transcriptional differences (Fig. 2B and Fig N in S1 Text). These inherent translational differences may come from codon usage, translation initiation, mRNA structure, and so on.

By contrast, the mRNA-RTE correlation of a single gene under multiple conditions reflects the relation between how the mRNA level and RTE change when the environment changes. We find both negative and positive mRNA-RTE correlations, and based on our significance analysis these are likely biologically meaningful (Fig E in S1 Text). For genes not sensitive to environmental changes, the mRNA level and RTE may be negatively correlated to stabilize protein production rate (Fig. 3A). For genes responding to specific nutrient limitations, the RTE may positively correlate with its mRNA level to amplify the change of overall protein synthesis rate, thus leading to a stronger correlation between mRNA level and protein abundance (Fig. 3B and Fig. 4). We again thank the reviewer for this insightful question. We have further explained the cross-gene and cross-condition correlations in the introduction in lines 68-71 and added more useful discussion in lines 535-538 to illustrate the importance of the cross-gene and cross-condition relation analysis.

4. It would be helpful if some statistics (like a p-value) could be provided to show the skewness of the distribution of the Spearman’s correlation of 2914 genes. (figure 2C)

We thank the reviewer for this suggestion. In the last version, we performed a statistical test on the two distributions, and the *p*-value between the two distributions was shown in Fig D in S1 Text. However, the reviewer's question reminds us that the Students’ *t*-test may not be suitable for this situation because the random RTE distribution is not strictly a normal distribution. Therefore, we changed to use the Kolmogorov-Smirnov test instead. According to the Kolmogorov-Smirnov test, the *p*-value between the two distributions is 5e-130. We have modified the legend in Figure 2C and Fig D in S1 Text accordingly.

5. The fold enrichment of the top 300 genes with the most negative correlation are generally weaker than the fold enrichment of the top genes with the most positive correlation (figure 3A and 3B). What could be the reason for this?

We thank the reviewer for the careful reading and for this insightful question. In order to answer this question, we first focus on the definition of fold enrichment:

$$Fold enrichment= \frac{GeneRatio}{BackgroundRatio}=\frac{g\_1/g\_input}{All\_1/All\_GO}$$

Where *g_input* is the number of genes we extracted for GO analysis, *g_1* is the number of genes annotated to the GO term in the input gene list for GO analysis, *All_1* is the number of all genes in the GO term, and *All_GO* is the number of all *E. coli* genes with at least one GO term. For the GO analysis shown in Figure 3A and 3B, *g_input* (g_input = 300 in our analysis) and *All_GO* are consistent. Therefore, we can rewrite this formula:

$$Fold enrichment= \frac{All\_GO}{g\_input}*\frac{g\_1}{All\_1}$$

The fraction before the asterisk is fixed, therefore, the difference in fold enrichment between the top 300 positive genes and top 300 negative genes, observed by the reviewer, comes from the fraction $\frac{g\_1}{All\_1}$.

We checked the *g_1* in our results, and we found little difference between the g_1 in the two clusters of genes, which is around 30. However, there are large difference between the values of All_1. For example, there are 234 genes involved in the regulation of RNA metabolic process, while there are only 10 genes involved in chemotaxis.

In fact, according to the paper (PMID: 30657448), bacteria such as *E. coli* have far more genes involved in metabolism than in environmental information processing. Indeed, as might be expected, the fold enrichment fluctuates more for GO terms with a small gene number. Therefore, one should not only use fold enrichment to determine whether a GO term is significantly enriched, but also pay attention to the *p*-value. In our study, the enrichment results were filtered with an adjusted *p*-value < 0.05 (See Methods, line 742), and the adjusted *p*-values were also shown in Fig. 3A-B.

6. Authors claimed that “pathways with similar translational regulation patterns tend to share similar codon usage” by comparing the codon frequencies of two pathways that have similar RTE variance across conditions or their mean RTEs were correlated. It would be helpful to see some control analysis:

1) How similar the codon usage would be for two randomly selected pathways?

2) Are there two pathways with related biological functionals but very different RTE variance pattern?

3) If there exist such pathways, do they share similar codon frequencies?

We thank the reviewer for these questions. First, it is difficult to directly compare randomly picked pathways: Gene often overlap among pathways, and the number of genes in some pathways is too small to support codon frequency analysis. Therefore, as a control analysis, we randomly selected gene sets containing 50 genes (about the average size of a pathway) and found that most of the correlation coefficients were in the range of -0.5~0.5, suggesting that the high correlation coefficients between the above true pathways are significant (Fig I(A) in S1 Text).

Statistically, we found that most of the KEGG pathways in our analyses had small RTE variance, less than 0.03 (Fig I(B) in S1 Text). Therefore, a few pathways with large RTE variance, i.e. with high variability in translation efficiency across nutrient limitations, may have biological relevance (Fig I(B) in S1 Text). We have added an analysis of the top 10 pathways with the highest RTE variance in Fig I in S1 Text. Interestingly, these pathways show generally high correlation coefficients between codon frequencies (Fig I(C) in S1 Text). This suggests that gene sets with high variability in translation efficiency under environmental stresses may share similar codon frequencies.

The four pathways we have shown in Figures 5 and 6 are typical examples that display similar RTE patterns across our tested range of nutrient limitations. However, different genes in a pathway, although functionally related, are not necessarily regulated in the same way. Therefore, the gene clusters that were selected based on RTE variance may not precisely match any individual pathway. For example, we found that the top 200 genes with the largest RTE variance in Figure 6A appeared in 45 out of 82 pathways. The top 200 genes with the smallest RTE variance in Figure 6A appeared in 42 out of 82 pathways, in both cases comprising a substantial fraction of *E. coli* pathways. Therefore, RTE variance may be an additional means by which translational regulation occurs, beyond the scope of pathway-specific regulation. We hope that the codon-usage-related RTE variability we identified may provide a useful guide for future work in this field.
